# Do Tonic Itch and Pain Stimuli Draw Attention towards Their Location?

**DOI:** 10.1155/2017/2031627

**Published:** 2017-12-07

**Authors:** Antoinette I. M. van Laarhoven, Stefaan van Damme, A. (Sjan) P. M. Lavrijsen, Dimitri M. van Ryckeghem, Geert Crombez, Andrea W. M. Evers

**Affiliations:** ^1^Health, Medical, and Neuropsychology Unit, Faculty of Social and Behavioral Sciences, Leiden University, Leiden, Netherlands; ^2^Leiden Institute for Brain and Cognition (LIBC), Leiden University, Leiden, Netherlands; ^3^Department of Psychiatry, Leiden University Medical Center, Leiden, Netherlands; ^4^Department of Experimental-Clinical and Health Psychology, Ghent University, Ghent, Belgium; ^5^Department of Dermatology, Leiden University Medical Center, Leiden, Netherlands; ^6^Faculty of Humanities and Social Sciences, Research Unit INSIDE, Institute of Health and Behaviour, University of Luxembourg, Esch-sur-Alzette, Luxembourg; ^7^Centre for Pain Research, University of Bath, Bath, UK

## Abstract

**Background:**

Although itch and pain are distinct experiences, both are unpleasant, may demand attention, and interfere with daily activities. Research investigating the role of attention in tonic itch and pain stimuli, particularly whether attention is drawn to the stimulus location, is scarce.

**Methods:**

In the somatosensory attention task, fifty-three healthy participants were exposed to 35-second electrical itch or pain stimuli on either the left or right wrist. Participants responded as quickly as possible to visual targets appearing at the stimulated location (ipsilateral trials) or the arm without stimulation (contralateral trials). During control blocks, participants performed the visual task without stimulation. Attention allocation at the itch and pain location is inferred when responses are faster ipsilaterally than contralaterally.

**Results:**

Results did not indicate that attention was directed towards or away from the itch and pain location. Notwithstanding, participants were slower during itch and pain than during control blocks.

**Conclusions:**

In contrast with our hypotheses, no indications were found for spatial attention allocation towards the somatosensory stimuli. This may relate to dynamic shifts in attention over the time course of the tonic sensations. Our secondary finding that itch and pain interfere with task performance is in-line with attention theories of bodily perception.

## 1. Introduction

Itch and pain are common somatosensory sensations, which, in acute form, function to protect body integrity, for example, penetration of the skin or stinging insects [1]. When chronic, for example, due to chronic inflammatory conditions of the skin, joints, or viscera, they often have a serious impact on quality of life and performance in daily activities [2–4]. One of the primary reasons for this burden is that itch and pain demand attention in order to perform their protective role [1, 5–7]. For example, when we touch a sharp object or red ants crawl on our skin, fast detection and identification of the threat along with interruption from a concurrent task are adaptive as we can impose action to prevent bodily damage. The interplay between attention and pain has frequently been investigated. The interplay between attention and itch, however, has barely received attention.

Leading cognitive frameworks on pain, which might to some extent also apply for itch, propose that pain draws attention and as such interrupts ongoing task performance and goal pursuit [7–12]. Overall, studies indicate that patients with chronic pain attend more to pain-related stimuli than control participants and have difficulties disengaging their attention away from pain [5, 6]. Such impaired ability to disengage attention from pain or pain-related information is believed to detrimentally affect functioning in daily activities [5–7]. Pain interferes with task performance [13–18], probably by directing attention to the location where the pain is expected and/or experienced. More recently, studies have focused upon the spatial attention allocation in pain [19–27]. It was found that attention was directed to the bodily location where threatening somatosensory stimuli were expected to occur [23–25]. It is reasonable to assume that individual differences in catastrophizing, worrying, and pain-related fear amplify the threat value of somatosensory stimuli and thus lead to a stronger prioritization of attention [5, 15, 28–32]. Also attempting to control pain leads to a similar allocation of attention towards the location where somatosensory stimuli are expected to occur [21, 26]. A heightened level of attention for pain and its location may then intensify the pain sensation or its impact upon daily functioning [5, 26]. These processes may also play a role in patients with chronic pain or itch [9, 10, 33, 34]. With regard to attention and itch, there are only some indications that itch-related information (e.g., words or pictures) draws attention [35–38] and that more bodily attention is related to heightened itch sensitivity [39]. However, research into spatial allocation of attention while experiencing itch is limited [38].

The investigation of spatial attention in pain and itch requires the use of specific paradigms. For example, spatial attention allocation has been investigated while participants perceive somatosensory pain stimuli on different locations while focusing on and responding to the location of tactile/visual/auditory target stimuli that are ipsilateral or contralateral to the pain location (e.g., [19–27]). Attention allocation to the stimulation location is inferred when participants respond faster to visual targets displayed ipsilaterally than on targets displayed contralaterally to stimulation, as can be deduced from the attentional bias index (i.e., the difference in response time to the contralateral minus the ipsilateral targets [20]). Enhanced focusing on the ipsilateral location is indicative for an attentional engagement, whereas faster responses on the contralateral location are indicative for disengagement of attention away from the stimulus, and when the attentional bias index significantly deviates from zero, there is an attentional bias. It has generally been found that pain draws attention towards its location, that is, attentional engagement [19–27]. Most of these studies, with the exception of [27], use phasic stimuli (≤1 s). However, patients often experience symptoms for a longer duration, stressing the importance of being able to disengage attention from pain and focus on activities in daily life. This is not only relevant for the study of pain, but also for itch, which is a sensation that is often prolonged by attentional processes, given its contagiousness [40]. For itch, we developed a somatosensory attention task (SAT) [38] with tonic itch stimuli of 35 s during which participants responded as quickly as possible to visual targets located at the stimulated or nonstimulated location. We did not find that healthy participants focused their attention towards the itch location; instead, we found some indications that participants disengaged their attention away from the itch location during the second half of the 35 s itch stimuli [38]. However, given the discrepancy with previous findings for pain showing that pain draws attention to its location, additional research involving both tonic itch and pain is required.

The aim of the present study was to investigate whether healthy participants focus their attention at or away from the tonic itch and pain stimulus location. It was expected that the participants' attention would be drawn to the location of the itch and pain stimuli early on but later on during the stimulation would disengage their attention from the stimulated location. Additionally, the relationship between attentional processing of itch and pain and other psychological characteristics, specifically self-reported catastrophizing, neuroticism, perceived threat of the somatosensory stimuli, attention for bodily sensations, and attentional disengagement from itch and pain was explored.

## 2. Methods

### 2.1. Participants

Fifty-three healthy volunteers (45 female/8 male; mean age of 22.0 years, SD = 2.2; range 18.6–29.4 years) were included. Participants were recruited through advertisements at Leiden University and the Leiden University Research Participation system (SONA systems Ltd., Tallinn, Estonia). Inclusion criteria for participation were being aged between 18 and 30 years (with the intention to include a homogenous group since reaction times increase with age [41]) and fluent in Dutch language. Exclusion criteria for participation were being a patient with chronic itch or pain, severe morbidity (e.g., multiple sclerosis, diabetes mellitus, heart or lung disease, and vasculitis), psychiatric disorders (e.g., depression), use of pacemaker, current use of medication (e.g., analgesics, antihistaminics), and pregnancy. Of the participants, 73.6% were following or had finished tertiary education, 24.5% were following or had finished secondary education, and 1.9% had followed primary education. The protocol was approved by the local Medical Review Ethics Committee and all participants provided written informed consent prior to testing.

### 2.2. Itch and Pain Induction

Itch and pain were induced electrically by means of a constant current stimulator (Isolated Bipolar Constant Current Stimulator DS5, Digitimer, United Kingdom) [37, 38, 42]. For itch induction, two surface electrodes were attached to the center of the lateral side of the wrist, a disk electrode (ø 1 cm, VCM Medical, The Netherlands) 1.5 cm proximal to the triquetrum, and a reference electrode (ø 2 cm, VCM Medical, the Netherlands) 2 cm proximal [37, 38, 42]. For pain induction, two surface electrodes (two disk electrodes of ø 1 cm, VCM Medical, the Netherlands) were attached at the center of the dorsal side of the wrist [20], one 1.5 cm proximal to the processus styloideus ulnae and the other 2 cm proximal. In accordance with our previous studies with electrically induced itch [37, 38, 42], the stimulus characteristics for the itch stimuli were 50 Hz frequency, 0.1 ms pulse duration, and a ramping of 0.05 mA/s. The itch stimuli lasted for at maximum 35 seconds, the duration of the stimuli in the SAT. For pain, the stimulus characteristics were partly based on previous studies (e.g., [24, 43]) and partly determined by extensive piloting of the methods since electrical pain stimuli are not regularly applied for 35 seconds. Eventually, pain stimuli were applied also at 50 Hz frequency and 0.4 ms pulse duration. Alike our previous studies [37, 38, 42], the maximum current for all stimuli was 5.00 mA. The levels of itch and pain evoked by each electrical stimulus were scored on a numerical rating scale (NRS) ranging from 0 (no itch/pain) to 10 (worst itch/pain ever experienced).

#### 2.2.1. Determination of the Intensity of the Itch Stimuli

In order to determine the individual intensity at which the 35 s baseline itch stimulus and the itch stimuli during the SAT were delivered, a step-up procedure was executed with 35 s stimuli starting at 0.25 mA, with 0.50 mA increments for every step. For example, the first stimulus started at 0.25 mA and, as a consequence of the ramping, ended at 2.00 mA; the second started at 0.75 mA and ended at 2.50 mA. Because the first step ended relatively high, just before the itch step-up, familiarization with the stimulation took place by assessing two perception thresholds starting at 0.01 mA and ending when the participant reported “the moment that you experience a sensation for the first time” [42]. The step-up procedure finished when the aimed NRS itch was at least 5 or the maximum defined current intensity of 5.00 mA was reached (i.e., stimulus from 3.25 to 5.00 mA). However, in the case the NRS itch exceeded 7, the current intensity was decreased with 0.5 mA (when NRS itch ≥ 8) or 0.25 mA (when NRS itch ≥ 7) up until the NRS itch was between 5 and 7. In this study, the determined starting current intensity for the baseline and SAT itch stimuli was on average 2.36 (SD = 1.26) mA.

#### 2.2.2. Determination of the Intensity of the Pain Stimuli

In order to determine the individual intensity at which the 35 s baseline pain stimulus and the pain stimuli during the SAT were delivered, a step-up procedure was executed with 10 s stimuli (in order to keep stimulation time better comparable to the itch step-up procedure which consisted of less steps) that increased by 0.50 mA per step. The first stimulus was given at 0.50 mA, the second at 1.00 mA, and so on. The step-up procedure was finished when the aimed NRS pain was at least 5 or the maximum defined current intensity of 5.00 mA was reached. However, in the case the NRS pain exceeded 7, the current intensity was decreased with 0.5 mA (when NRS pain ≥ 8) or 0.25 mA (when NRS pain ≥ 7) up until the NRS pain was between 5 and 7. In this study, the determined current intensity for the 35 s baseline pain stimulus before the SAT and the pain stimuli during the SAT was on average 3.70 (SD = 1.59) mA.

### 2.3. Somatosensory Attention Task

**Figure 1:**
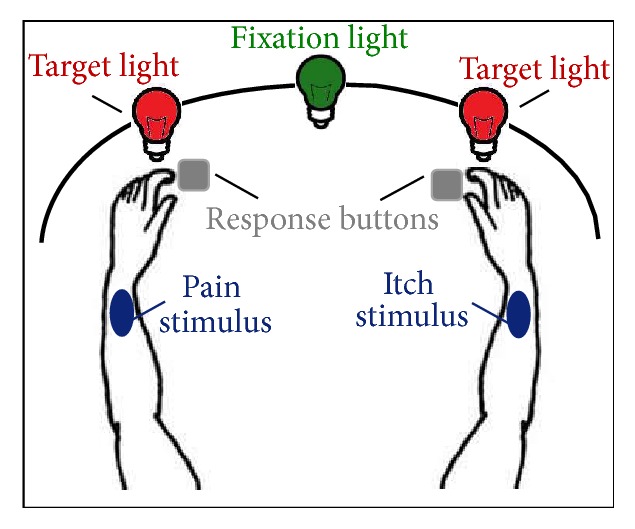
Schematic representation of the setup of the somatosensory attention task. The side of itch stimulation was contralateral to the pain stimulation (randomized across participants). During a block, an itch (itch block) or pain (pain block) stimulus was applied, or no stimulation (control blocks), while, after short onset of the fixation light, one of the target lights illuminated. Participants responded to the target light location using response buttons right below both target lights, at either the ipsilateral or the contralateral location as opposed to the somatosensory stimulation.

The somatosensory attention task (SAT) as used in our previous study [38], which was based on an attention task developed for pain [20], was adopted to investigate attention allocation towards both an itch and pain stimulation and their location (see Figure 1 for a schematic representation of the setup). A plastic black curved screen of ca. 50 cm height with 3 LED lights at 10 cm height (middle green fixation LED, the left and right were red target LEDs placed at 25 degrees from the middle LED) was placed in front of the participant. The LEDs were controlled using E-prime software version 2.0 (Psychology Software Tools Inc., Sharpsburg, PA, USA) on a Dell optiplex 3010 computer with Philips Brilliance 225 TFT screen (Resolution 1280 × 1024 at 60 Hz). Right below the left and right LED there was a platform with finger response buttons (Pushbutton Switch, SPDT, Off-(On)) at a fixed position, attached to a serial response box (Psychology Software Tools Inc. Sharpsburg, PA, USA).

The SAT consisted of 12 blocks of 35 seconds each, of which 4 blocks with pain stimuli (pain blocks), 4 blocks with itch stimuli (itch blocks), and 4 blocks without somatosensory stimulation (control blocks). The order of blocks was randomized by E-Prime for each participant. The standard interval between two blocks was 1 minute, which was extended by 1 minute up to a maximum of 5 minutes in the case the NRS pain or NRS itch exceeded 2.0. During each block 10 trials with visual targets were administered, in which first the fixation light (green LED light) was turned on for 1000 ms and extinguished, and then either the left or right target (red LED light) was turned on for 200 ms [38], while unilaterally administering itch (itch blocks) or pain (pain blocks) stimuli, or no stimulation (control blocks). The response window for participants to press a button was 1500 ms. The 10 target stimuli in each block were given in random order with random time interval (varying between 0 and 2000 ms) before the next trial. Half of the visual targets were presented at the wrist where the electrodes were attached and itch or pain was applied in the case of itch and pain block, respectively (“ipsilateral trials”), and half of the visual targets were presented oppositely (“contralateral trials”). Conforming previous research (e.g., [20]), the difference in participants' responding to ipsilateral versus contralateral trials is a measure of spatial attention allocation towards the somatosensory stimulus, with faster responses to ipsilateral trials being indicative for an attentional bias.

### 2.4. Self-Report Questionnaires

The following self-report questionnaires were administered in Dutch using the online system Qualtrics (Provo, Utah, USA).

The* presence of physical symptoms* was assessed by visual analogue scales (VAS) for itch and pain from the Impact of chronic skin disease on daily life (ISDL) [44], inquiring about the levels of itch and pain during the past two weeks on a scale from 0 (no itch/pain) to 10 (worst itch/pain experienced).

*Psychological distress* was measured with the* Hospital Anxiety and Depression Scale (HADS) [45]* and a short version of the* Positive and Negative Affect Schedule (PANAS) *[46]. The HADS consists of 7 items measuring the subscale depression (Cronbach alpha in the present study was 0.67) and 7 items measuring the subscale anxiety (Cronbach alpha 0.71), scored on a scale from 0 to 3. The total score was obtained by summing the items per subscale. The PANAS consists of 5 positive items (PANAS-PA; Cronbach alpha 0.59) and 5 negative items (PANAS-NA; Cronbach alpha 0.35) scored on a 5-point Likert scale from 1 to 5. Due to the low reliability, the PANAS was excluded from data analyses.

*Catastrophizing* about physical sensations was measured using the* Pain Catastrophizing Scale* [47], adjusted for physical sensations (PCS-A) in order to make it also applicable to itch (i.e., by substituting the word “pain” for “physical sensations” for all concerning items). The questionnaire contained 13 items, which were scored on a 5-point Likert scale from 0 to 4. The Cronbach alpha for the PCS-A in the present study was 0.87.

*Neuroticism* was measured with the* Eysenck Personality Questionnaire revised short scale* (EPQ-RSS) [48], consisting of different subscales, including the subscale neuroticism (Cronbach alpha = 0.72), which consists of 12 items rated on a dichotomous scale (yes = 1/no = 0).

*Fear of pain *was measured using the Fear of Pain Questionnaire III (FPQ-III) [49], with 30 items assessing the degree of fear participants would likely experience in potentially painful situations, subdivided in the categories severe pain, minor pain, and medical pain. The items are rated on a 5-point scale from 1 (not at all fearful of this pain) to 5 (extremely fearful of this pain). Cronbach alpha of the FPQ-III in the present study was 0.90.

*Attentional focus on bodily sensations* was measured using the Body Vigilance Scale (BVS) [39, 50], the* Body Sensations questionnaire* [39, 51], and the* Pain Vigilance* and Awareness Questionnaire [52] adjusted for physical sensations (i.e., by substituting the word “pain” by “physical sensations” for all concerning items)* (PVAQ-A)* in order to make it broadly applicable to physical sensations, including itch and pain. The BVS, used to measure attentional focus on bodily sensations, contained 4 items, of which the fourth item consisted of 13 subitems about anxiety-related bodily sensations. All items were rated on a VAS from 0 to 10. Cronbach alpha of the BVS in the present study was 0.79. Additionally, two items had been added that assess one's attention directed towards itch and pain. Of the BSQ, the 15 items concerning bodily sensations (omitting the 2 items concerning derealization) were used to measure of attentional focus on the occurrence of bodily sensations when in a nervous or feared situation (e.g., heart palpitations, dizziness or sweating). Participants used a 5-point Likert scale that ranged from “the sensation never occurs” (0) to “the sensation occurs almost always or always” (4). Cronbach alpha of the BSQ in the present study was 0.79. The PVAQ-A was used to measure attention to bodily sensations by asking subjects to consider their behavior in relation to physical sensations. The PVAQ-A (Cronbach alpha 0.85) consisted of 16 items, for example, “I focus on physical sensations.” Items were scored on a 6-point Likert scale (0 never to 5 always).

*Attentional disengagement from itch and pain *was assessed using two Likert scales ranging from 1 (not at all able to disengage attention) to 5 (always able to disengage attention).

In addition to these online questionnaires, participants indicated the perceived threat of the stimuli applied in the experiment on a scale from 0 (not threatening) to 10 (very threatening). Participants also rated the extent to which they were distracted by the itch or pain stimuli or other factors during their responses to the visual targets in the SAT on 5-point Likert scales ranging from 1 (not at all distracted) to 5 (distracted to very large extent).

### 2.5. Procedure

Potential participants were informed about the study via written information. When interested in participation, they clicked on an online link to fill out several questions. These concerned demographic variables, absence or presence of medical or psychiatric conditions, intake of medication during the past 4 weeks, the VAS for itch and pain, HADS, PCS-A, EPQ-RSS, FPQ-III, BSQ, PVAQ-A, and attentional disengagement from itch and pain (see Section 2.4). Based on the online assessment, eligibility screening was performed on inclusion and exclusion criteria. Uncertainties about eligibility were solved by telephone contact. Eligible participants made an appointment for participation. Participants were instructed to refrain from intake of alcohol and drugs 24 hours before attending the experiment. Upon arrival at the test facility, participants were verbally informed about the procedure and told that they were free to terminate the experiment at any time. Then participants signed the informed consent. In the lab, subjects also rated their current levels of spontaneous itch and pain as well as perceived fatigue on an NRS ranging from 0 (no itch/pain/fatigue) to 10 (worst itch/pain/fatigue ever experienced) and filled out the BVS and PANAS.

In order to standardize the participants' wrist temperature, which could influence electrical conductivity [53], subjects held their wrists for 3 minutes in a warm water bath made at 34°C [see also [37, 42]], before the electrical stimulation. The side of itch and pain stimulation (left and right wrist or vice versa) was randomized across participants. Then, the step-up procedures for itch and pain were carried out in random order to determine the individual intensity of the itch and pain stimuli. At the individually determined intensity, baseline itch and pain stimuli were applied for 35 seconds. Right before the SAT, participants were asked to position their index fingers of the left and right hand on the left and right response button, respectively. They were instructed to focus on the visual stimuli and to respond as quickly as possible to the location of a target LED illuminating, by pressing the response button at the ipsilateral side. Before each block, participants were informed whether they would receive a pain stimulus (i.e., pain block), an itch stimulus (i.e., itch block), or no stimulus at all (i.e., control block). At the start of each block, the experimenter counted down from 3 to 0, to indicate the onset (at 0) of a block. Directly following each block, participants were asked to retrospectively report the levels of itch and pain that were evoked (irrespective of any ongoing spontaneous itch or pain) during the block on NRSs ranging from 0 (no itch/pain) to 10 (worst itch/pain ever experienced). After all measurements, participants indicated the perceived threat of the itch and pain stimuli and the extent to which they were distracted during their task performance to respond to the visual targets. After a short debriefing, participants received a monetary reimbursement.

### 2.6. Statistical Analyses

Reaction times (RT) for trials with RT ≥ 150 ms (0.2% of the trials were excluded) and trials with correct responses (0.6% of the trials were excluded) were extracted from E-prime. Data of two participants were excluded [fire alarm evacuation (*n* = 1), problems with itch stimulation (*n* = 1)] because ≤70% of the RT data was available [38]. Using Matlab and Statistics Toolbox Release 2012b (The MathWorks, Inc., Natick, Massachusetts, USA) the mean RT per trial type (i.e., ipsilateral and contralateral trials during pain, itch, and control blocks) were calculated per participant. Participants' accuracy for the SAT was checked, and no one's data had to be removed based on the criterion of >30% mistakes [38]. Additionally, RT per trial type were calculated for three consecutive time segments of the 35 s SAT blocks. Three was the maximum number of segments the blocks could be split into to remaining sufficient observations per trial type.

All variables to be included in the statistical analyses were checked for normal distribution and transformed when necessary. Transformation did not result in normal distribution of the NRS itch and pain scores during the control blocks and assumptions for the majority of psychological characteristics were not met. In addition, there were two participants displaying outlying RT (i.e., >3 SD of the overall mean) for the majority of the trial types. Therefore, the analyses were conducted both in all 51 participants and after excluding the two outliers (*n* = 49) combined with log-transforming variables.

A manipulation check, to confirm that the intended sensations had been induced in the respective blocks, was conducted comparing the NRS itch and pain scores for the itch and pain blocks, respectively, to the control blocks using nonparametric sign tests. Similarly, NRS unpleasantness ratings were exploratorily compared across the different block types. An attentional bias index (AB-index) was calculated for itch and pain [20] using the formula RT_contralateral_ − RT_ipsilateral_, during itch and pain blocks, respectively. A positive AB-index indicated that attention was directed ipsilaterally to the stimulus location (attentional engagement), while a negative AB-index indicated that attention was directed contralaterally to the stimulus location (attentional disengagement). One-sample* t*-tests were conducted to assess whether the AB-indices significantly differed from zero, that is, implying attentional bias. In order to test the main hypothesis of whether participants focused attention on the itch and pain location, two repeated measures analyses of variance (RM-ANOVAs) were carried out with the within-subjects factors location (ipsilateral versus contralateral) and block type (either itch or pain versus control). Separate analyses for itch and pain were required because the factor location in the control blocks referred to the location of the attached itch and pain electrodes, which were oppositely attached, and, consequently, for control blocks, the ipsilateral location was indecisive. Main effects of location and block type were calculated, as well as location × block type interactions. Exploratorily, a similar RM-ANOVA was conducted to compare the RT for the itch versus pain blocks (control blocks were not included). In order to investigate the course of attention allocation over time, 2 × 2 × 3 RM-ANOVAs were conducted, for itch and pain separately, with the within-subjects factors location (ipsilateral versus contralateral), block type (either itch or pain vs. control), and time (first, second, and third time segment of blocks). Main effect of time and location × block type × time interactions were calculated. For all RM-ANOVAs, a generalized eta squared was calculated [54, 55].

Finally, Pearson correlation coefficients were calculated between the AB-indices for itch and pain. Nonparametric correlation coefficients (Spearman) were calculated between the psychological characteristics (EPQ-RSS-n, BVS, BSQ-f, PVAQ-A, PCS-A, FPQ-III, attentional focus on and disengagement from itch and pain, and perceived threat of the stimuli) and itch and pain AB-indices.

Statistical analyses were conducted using SPSS 23.0 software (IBM SPSS Statistics for Windows, Armonk, NY, USA). All values displayed are means ± SD, unless stated otherwise. A *p* < 0.05 was considered statistically significant.

## 3. Results

### 3.1. Participants

The baseline levels of itch, pain, and fatigue and outcomes of self-report questionnaires measuring the psychological characteristics of the 53 participants included are displayed in Table 1. The reasons for baseline spontaneous itch levels >0 (*n* = 10 in total, M_NRS-itch>0_ = 1.1 ± 0.5, ranging from 0.5 to 2.0) were talking/thinking about itch as a result of this specific question (*n* = 5), dry skin (*n* = 2), sweating due to traveling (*n* = 1), epilated armpit (*n* = 1), and some skin irritation (*n* = 1). The reasons for baseline spontaneous pain levels >0 (*n* = 8 in total; *M*_NRS-pain>0_ = 1.1 ± 0.5, ranging from 0.3 to 2.0) were sore throat (*n* = 2), muscle ache (*n* = 2), back ache (*n* = 1), knee pain resulting from surgery some weeks ago (*n* = 1), menstruation pain (*n* = 1), and finger cut (*n* = 1).

### 3.2. Manipulation Check: Induced Itch and Pain

The itch, pain, and unpleasantness scores for the baseline itch and pain stimuli and those during the SAT blocks are displayed in Table 2. Nonparametric sign tests showed that median NRS itch scores were significantly higher during itch than control blocks of the SAT and median NRS pain scores were significantly higher during pain than control blocks (both *p* < 0.0005). Median NRS unpleasantness scores were significantly higher during itch and pain blocks than during control blocks (both *p* < 0.0005) and also significantly higher during pain blocks than during itch blocks (*p* < 0.0005).

### 3.3. Perceived Threat of the Stimuli

The induced pain and itch were, on average, perceived as 2.8 ± 2.4 and 1.5 ± 1.8 threatening, respectively. With regard to the degree to which participants were distracted from the task to respond to the visual targets, they indicated to be distracted by the itch and pain stimuli on average 3.2 ± 1.0 and 2.5 ± 1.1, respectively, and 1.8 ± 0.6 by other factors.

### 3.4. Behavioral Outcomes

With regard to the accuracy, the average number of mistakes made during the SAT over all participants was 0.6 ± 1.3 (range 0 to 8; theoretical maximum 120), with overall 0.5% mistakes during itch blocks, 0.4% mistakes during pain blocks, and 0.6% mistakes during control blocks. The mean RTs (of correct responses) during itch, pain, and control blocks for the ipsilateral and contralateral trials are displayed in Table 3.

The location × block type interaction effect was of primary interest to this study as this indicated whether attention was drawn to the stimulus location. For itch, the RM-ANOVA comparing the ipsilateral and contralateral trials (factor 1: location) during the itch and control blocks (factor 2 block type) did not show a significant location × block type interaction effect (*F*(1,50) = 0.78, *p* = 0.38, *η*_G_^2^ = 0.0014). There was, however, a significant main effect of block type (*F*(1,50) = 12.80, *p* < 0.001, *η*_G_^2^ = 0.019), with longer RT for itch blocks than control blocks. The main effect of location was not significant (*F*(1,50) = 0.13, *p* = 0.72, *η*_G_^2^ = 0.0003). For pain, the RM-ANOVA did not show a significant interaction effect of location × block type (*F*(1,50) = 0.71, *p* = 0.41, *η*_G_^2^ = 0.00012). Again, there was a significant main effect of block type (*F*(1,50) = 21.29, *p* < 0.0001, *η*_G_^2^ = 0.05), with longer RT for pain blocks than for control blocks, but no significant main effect of location (*F*(1,50) = 0.16, *p* = 0.69, *η*_G_^2^ = 0.00032). After removing the two outliers, similar levels of significance were obtained. In line with the main findings of the nonsignificant location × block type interaction, no significant attentional biases were found as the AB-indices for itch (*t*(50) = −0.51, *p* = 0.61) and pain (*t*(50) = 0.18, *p* = 0.86) did not significantly differ from zero.

Explorative comparison of the itch and pain blocks showed no significant interaction effect of location × block type (*F*(1,50) = 0.13, *p* = 0.72, *η*_G_^2^ = 0.00036), nor a significant main effect of location (*F*(1,50) = 0.004, *p* = 0.952, *η*_G_^2^ = 0.00001), but the overall RT were significantly longer for the pain than for the itch blocks (*F*(1,50) = 5.26, *p* = 0.026, *η*_G_^2^ = 0.0109).

### 3.5. Time Course of Attention during the SAT

**Figure 2:**
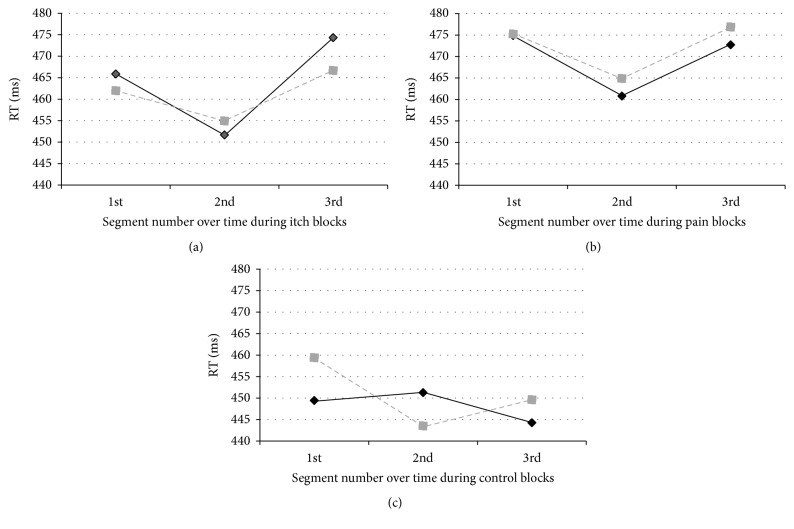
Reaction times (in ms) for participants (*n* = 51) responding to the visual target lights during the 35 s somatosensory itch (a) or pain bocks (b) or in control blocks, in which no somatosensory stimulation was applied (c). Visual targets were displayed either at the side of the itch or pain stimulation (i.e., ipsilateral trials, solid black line) or at the opposite side (i.e., contralateral trials, dashed grey line). In the case of control blocks, the solid black line is indicative for trials ipsilaterally to the attachment of the itch electrodes and the dashed grey line is indicative for trials ipsilaterally to the attachment of the pain electrodes.

In a further analysis of the data, Figure 2 displays the RT for the ipsilateral and contralateral trials during the itch (Figure 2(a)), pain (Figure 2(b)), and control (Figure 2(c)) blocks, which are subdivided into three equal time segments. For itch, there was no significant location × block type × time interaction (*F*(2,100) = 2.01, *p* = 0.140, *η*_G_^2^ = 0.0068), but a significant main effect of time (*F*(2,100) = 3.77, *p* = 0.026, and *η*_G_^2^ = 0.015) emerged. Simple contrast analyses showed that RT were significantly faster in the second than in the first segment (*F*(1,50) = 6.73, *p* = 0.012 and, *η*_G_^2^ = 0.006). There were no significant differences in RT when comparing the second with the third segment, although a nonsignificant trend was observed (*F*(1,50) = 4.03, *p* = 0.050, and *η*_G_^2^ = 0.038), or when comparing the first and the third segment (*F*(1,50) = 0.48, *p* = 0.494, and *η*_G_^2^ = 0.0094). For pain, there was no significant location × block type × time interaction (*F*(2,100) = 0.41, *p* = 0.662, and *η*_G_^2^ = 0.0012), nor a significant main effect of time, although a trend was observed (*F*(2,100) = 2.99, *p* = 0.055, and *η*_G_^2^ = 0.012).

After removing the two outliers, similar results were obtained in the 2 × 2 × 3 RM-ANOVA for itch. For pain results were also comparable after removing the two outliers, although now a significant main effect of time (*F*(2,96) = 3.17, *p* = 0.047, and *η*_G_^2^ = 0.015) was found. Simple contrast analyses showed significantly faster RT in the second than in the first segment (*F*(1,48) = 7.30, *p* = 0.010, and *η*_G_^2^ = 0.026), but no significant differences in the second compared to the third segment (*F*(1,48) = 1.43, *p* = 0.237, and *η*_G_^2^ = 0.011) nor in the first compared to the third segment (*F*(1,48) = 1.54, *p* = 0.221, and *η*_G_^2^ = 0.019).

### 3.6. Exploratory Analyses: Association between Individual Characteristics and Attentional Bias towards Itch and Pain

The AB-index for itch was on average −2.9 ± 39.9 and ranged from −80.1 to 90.2; 39.2% of the participants displayed a positive AB-index (i.e., towards the itch stimulus location). The AB-index for pain was on average 1.0 ± 41.0 and ranged from −79.5 to 86.5; 54.9% of the participants displayed a positive AB-index (i.e., towards the pain stimulus location). The AB-indices for itch and pain were not significantly correlated (*R* = −.252, *p* = 0.074). The AB indices were generally not significantly correlated with the psychological characteristics neuroticism (EPQ-RSS), catastrophizing of physical sensations (PCS-A), fear of pain (FPQ-III), self-reported attention to itch and pain and to bodily sensations in general (BVS, BSQ, PVAQ-A), attentional disengagement from itch and pain, and the perceived threat of the induced itch and pain. Only four significant correlations were observed. There were positive associations between the AB-index for itch and catastrophizing (*r*_*S*_ = 0.40, *p* = 0.003), neuroticism (EPQ-RSS-n) (*r*_*S*_ = 0.37, *p* = 0.008), and the threat value of the itch stimulus (*r*_*S*_ = 0.29, *p* = 0.04). There was a negative association between the AB-index for pain and the threat value of the pain stimulus (*r*_*S*_ = −0.30, *p* = 0.03).

## 4. Discussion

The present study investigated whether attention of healthy volunteers would be spatially drawn to the stimulus location early on during tonic itch and pain stimuli, and, whether they would disengage their attention away from the stimulated location later on during stimulation. In the somatosensory attention task, participants received tonic somatosensory itch or pain stimuli or no stimulation while responding to the location of visual targets, either ipsi- or contralaterally displayed to the somatosensory location. In contrast with our expectations, no significant differences were found between responding to visual targets ipsilaterally compared to contralaterally to the stimulation, neither over the total duration of stimulation nor across the three successive time segments during the tonic itch and pain stimuli. Of further note, we observed that itch and pain stimulation slowed down participants' task performance (i.e., responding to visual targets) compared to no stimulation, indicating towards attentional interference by itch and pain. Overall, these results seem to indicate that itch and pain affect attentional processes, but that attention is not systematically directed towards nor disengaged from the location of tonic itch and pain stimulation.

There were no indications that attention was directed away from or towards the location of the itch and pain stimulation: reaction times for ipsilateral and contralateral trials did not significantly differ, nor was there a significant difference in spatial attention allocation between itch and pain. The indications for an attentional disengagement effect during the last part of the 35 s itch stimulation in our previous study [38] could not be confirmed here. In addition, we were also not able to replicate previous findings that pain directs attention towards its spatial location [19–27]. However, most of these studies used phasic pain stimuli with each trial consisting of one pain stimulus and one target stimulus [19–26] or pain stimuli of maximally 10 seconds [27]. It could be that the 35 s somatosensory stimuli in the present study along with multiple trials of visual targets during that stimulus may not draw attention to the stimulus location for the entire time frame. Attention likely continuously shifted between the somatosensory stimuli and visual targets. This process may have been enhanced because the participants were aware that the visual targets could be displayed ipsilateral or contralateral to the stimulation and the central fixation light before each trial could have influenced attention allocation. Moreover, the intensity of the itch and pain stimuli and the threatening character of the stimuli were relatively mild, and therefore the stimulus saliency may have been limited. Generally, in the present and the previous study there was a time effect showing that participants responded faster after the first segment. This may be owing to a learning effect as the participants learned to respond faster to the visual targets, leaving less attention to focus on the itch and pain sensations. This effect was, however, irrespective of the spatial location of the somatosensory stimuli. It could be that somatosensory stimuli only draw attention to the spatial location in the very beginning. However, the current segmentation of three time segments might not be sufficiently fine-grained to determine continuous attentional shifts.

Of further note, our study did show that participants were generally slower in task performance of responding to the targets during itch and pain, which is indicative for attentional interference by itch and pain. The fact that pain interferes with attention has previously been demonstrated [13–18] although most studies used stimuli with a duration shorter than 35 s. Surprisingly, in our previous study with itch stimuli similar to those in the present study we did not find such an interference effect [38]. Exploratory findings indicate that pain may interfere more in attentional processing than itch, as overall reaction times (i.e., independent of stimulus location) were slower during pain than during itch. Explanations for this may include that pain is evolutionarily more aversive, as indicated by the higher reported threat value and unpleasantness of the pain stimuli presented here and, consequently, a higher saliency [10, 12]. However, it could also be related to the lower levels of evoked itch than pain. Reversely, participants may have better been able to ignore the itch and therefore perceived itch less intense during the attention task, akin previous findings showing that focusing away from pain can result in less intense pain [27, 56]. Support for this explanation comes from the large decline in itch when comparing the itch stimuli, at the same intensity, given at baseline and during the attention task. Another possible explanation could be that people habituate more easily to itch than to pain, but this has, to our knowledge, not yet been investigated.

Of the psychological characteristics the individual levels of catastrophizing of physical sensations and neuroticism were related to a higher attentional bias index for itch. However, given the nonsignificant association between catastrophizing and the attentional bias index for pain, these findings should be interpreted with caution. There were also some indications that higher perceived threat of the itch stimulus was related to a higher attentional bias index for itch, but higher perceived threat of the pain stimulus was associated with a lower attentional bias index for pain, which is contrary to what would be expected. Other psychological characteristics, including fear of pain and self-reported attention to and disengagement from physical sensations and itch and pain in particular, did not play a role in attention allocation towards the itch and pain stimuli. Future research should further investigate the role of individual characteristics in spatial attention allocation towards itch and pain.

This study has several limitations. First, the levels of itch induced during the attention task were relatively low and not directly comparable to pain. Second, after each block in the SAT, participants retrospectively rated the intensity of itch and pain during the somatosensory stimulation. It cannot be ruled out that participants also intentionally focused on the stimulation while responding to the visual targets. Third, the current design did not allow the investigation of fast attentional switches between somatosensory and visual stimuli. Future research may use more fine-grained time segments. Fourth, the included group was homogenous with respect to age but this limits extrapolation to other age groups.

## 5. Conclusions

This study showed that although tonic itch and pain stimuli interfere with task performance, attention is not consistently drawn towards their spatial location, probably because attention shifts over the time course of tonic stimuli. Additional research focusing more closely on time aspects of attention allocation is required to elucidate how tonic itch and pain stimuli are being processed in healthy participants and in clinical populations. When focusing attention on the location of itch or pain aggravates symptoms, patients with chronic itch and pain may benefit from learning to disengage their attention away from itch or pain, respectively.

## Figures and Tables

**Table 1:** Total scores of self-reported questionnaires (*n* = 53).

	Mean score ± SD	Range
Level of spontaneous itch at baseline	0.2 ± 0.5	0.0–2.0
Level of spontaneous pain at baseline	0.2 ± 0.4	0.0–2.0
Level of fatigue at baseline	1.8 ± 1.3	0.0–5.5
Affect		
Anxiety (HADS-Anxiety)	2.4 ± 0.5	0.9–3.0
Depression (HADS-Depression)	2.7 ± 0.3	1.9–3.0
Personality characteristics		
Neuroticism (EPQ-RSS)	3.2 ± 2.5	0–11.0
Attention to bodily sensations		
Attentional focus on itch	2.2 ± 1.9	0–6.5
Attentional focus on pain	3.3 ± 2.4	0–8.0
BVS	2.8 ± 1.5	0.2–6.8
BSQ	2.0 ± 0.5	1.3–3.3
PVAQ-A	24.2 ± 9.5	4–45
Catastrophizing		
PCS-A	7.5 ± 6.4	0–29
Fear of pain		
FPQ-III	63.3 ± 15.9	36–101
Attentional disengagement from		
Itch	4.3 ± 1.0	1–5
Pain	4.0 ± 0.9	1–5

HADS: Hospital Anxiety and Depression Scale (theoretical range 0–21 per subscale); EPQ-RSS: Eysenck Personality Questionnaire revised short scale (theoretical range 0–12 neuroticism subscale); Single items assessing attentional focusing on itch and pain (theoretical range 0–10); BVS: Body Vigilance Scale (theoretical range 0–10); BSQ: Body Sensations Questionnaire (theoretical range 1–5); PVAQ-A: Pain Vigilance and Awareness Scale, adjusted for physical sensations (theoretical range 0–80); PCS-A: Pain Catastrophizing Scale, adjusted for physical sensations (theoretical range 0–52); FPQ: Fear of pain questionnaire (theoretical range 30–150); single items about attentional disengagement (theoretical range 1–5).

**Table 2:** Means ± standard deviations of NRS itch, pain, and unpleasantness scores at baseline and during the pain, itch and control blocks of the somatosensory attention task (SAT) (*n* = 51).

	NRS itch	NRS pain	NRS unpleasantness
Baseline itch stimulus	**3.5 ± 2.2**	0.6 ± 1.1	2.3 ± 2.0
Baseline pain stimulus	0.9 ± 1.3	**3.9 ± 1.7**	3.4 ± 1.8

SAT itch blocks	**1.8 ± 1.6**	0.2 ± 0.4	1.2 ± 1.5
SAT pain blocks	0.5 ± 0.8	**3.0 **± **1.7**	2.7 ± 1.7
SAT control blocks	0.1 ± 0.2	0.0 ± 0.1	0.0 ± 0.1

*Note*. The electrical current at which the itch and pain stimuli were applied was tailored to individual sensitivity and was identical during baseline measurements and the SAT. NRS: numerical rating scale.

**Table 3:** Mean reaction times (in ms) ± standard deviation for the ipsilateral and contralateral trials of the somatosensory attention task (SAT) during itch, pain, and control blocks (*n* = 51).

	Mean reaction times (ms) of ipsilateral trials	Mean reaction times (ms) of contralateral trials
Itch blocks	466.2 ± 91.0	463.7 ± 84.4
Pain blocks	470.7 ± 81.8	472.5 ± 80.9
Control blocks	450.4 ± 81.2^1^	457.4 ± 88.5^2^

^1^Reaction times during control blocks (no somatosensory stimulation) ipsilateral to attached itch electrodes location. ^2^Reaction times during control blocks (no somatosensory stimulation) ipsilateral to the attached pain electrodes location.
